# Therapeutic role of *Crateva religiosa* in diabetic nephropathy: Insights into key signaling pathways

**DOI:** 10.1371/journal.pone.0324028

**Published:** 2025-05-28

**Authors:** Muhammad Ali, Hafiz M. Irfan, Alamgeer, Aman Ullah, Magda H. Abdellattif, Mahmoud Elodemi, Mohammad Zubair, Ajmal Khan, Ahmed Al-Harrasi

**Affiliations:** 1 College of Pharmacy, University of Sargodha, Sargodha, Pakistan; 2 Punjab University College of Pharmacy, University of the Punjab, Lahore, Pakistan; 3 Department of Pharmacy, Saba Medical Centre, Abu Dhabi, United Arab Emirates; 4 Chemistry Department, College of Sciences, University College of Taraba, Taif University, Taif, Saudi Arabia; 5 Department of Pharmacology, Faculty of Medicine, University of Tabuk, Tabuk, Saudi Arabia; 6 Department of Medical Microbiology, Faculty of Medicine, University of Tabuk, Tabuk, Saudi Arabia; 7 Natural and Medical Sciences Research Center, University of Nizwa, Nizwa, Oman; 8 Department of Chemical and Biological Engineering, College of Engineering, Korea University, Seoul, Republic of Korea; Helwan University, EGYPT

## Abstract

*Crateva religiosa*, a plant used in traditional medicine, is valued for its bioactive properties. Traditional approaches are more accepted worldwide as a cost effective alternatives being used in network pharmacology to explore the complex interactions of drug targets among molecular pathways. The study investigated the potential of Crateva religiosa’s phytoconstituents using meticulous computational analysis and empirical confirmation. The IMPPAT, GeneCards and DisGeNET data bases were used to obtain the active moieties and disease targets respectively. Crateva phytoconstituent’s DN-target network and protein-protein interaction (PPI) network were developed and analyzed using the STRING online platform and Cytoscape software. GO and KEGG analyses were conducted using the g: profiler databases while the process of molecular docking involved the use of MOE software. The screening process identified dillapiole (CR-C1), beta ionone (CR-C2) 10-epi-γ-eudesmol (CR-C3), cis/trans linalool oxide (CR-C4/5) and nerolidol (CR-C6), as potential active phytoconstituents of *C. religiosa* and AKT1, PPARG, PTGS2, EGFR, ESR1, JAK2, MAPK1, PARP1, GSK3B, and PPARA as matching targets in DN. The enrichment analysis revealed that the common targets were primarily linked to inflammatory response, oxidative stress, immunological modulation, and cell death. The main signal pathways suggested were PI3K-Akt, AGE-RAGE, and IL-17. Moreover, molecular docking analysis determined that the AKT1, PPARG and PTGS2 are the essential targets that had a good affinity for their respective active molecules.

## 1. Introduction

Diabetes mellitus, including types 1 and 2, has heavily burdened public health over the past few decades due to its high mortality rate and challenging treatment regimen [1,2]. In 2021, prevalence of diabetes among adults aged 20–79 worldwide was 10.5%, affecting more than 536 million people. By 2045, the number is expected to increase to 12.2%, affecting 783 million people [3].

The dynamic progression of diabetes complications has unfortunately led to numerous attempts at treatment and prevention. Of these, the most prevalent complication of diabetes is diabetic nephropathy (DN), also known as diabetic kidney disease (DKD). A significant burden on public health results from the fact that at least 30% of diabetes patients have DN [4]. According to the most recent data, from 1997 to 2017, DN was responsible for one-third of the disability-adjusted life-years of all patients with chronic renal disease worldwide [5].

In the majority of developed nations, DN is now the primary cause of chronic kidney disease and renal failure [6,7]. Nevertheless, despite stringent blood pressure and glucose control protocols, the incidence of DN is on the rise [8]. Thus, oxidative stress, autophagy, and protracted microinflammation are only a few of the several other pathways that contribute to the pathophysiology of DN [9].

Angiotensin receptor blockers (ARBs) have demonstrated efficacy in the treatment of diabetic nephropathy (DN). According to the findings of the IDNT (Irbesartan Diabetic Nephropathy Trial) trial, it was observed that irbesartan exhibited a 20% reduction in the risk of the primary composite endpoint, which includes the doubling of baseline serum creatinine concentration, onset of end-stage renal disease (ESRD), or mortality [10]. Recent research findings indicate that sodium-glucose cotransporter-2 inhibitors (SGLT2is), a novel class of hypoglycemic agents, have favorable outcomes in the treatment of diabetic nephropathy (DN) [11–13]. Nevertheless, the therapeutic applicability of both ARBs and SGLT2is is limited to some extent due to the presence of adverse effects. ARBs can cause a decrease in glomerular filtration rate (GFR) and an increase in serum potassium levels. If the serum creatinine exceeds 3 mg/dl, ARBs are not appropriate. In addition, it is worth noting that SGLT2is might potentially lead to some negative consequences, such as the development of mycotic vaginal infections [14], diabetic ketoacidosis [15], and lower extremity amputation [16].

Plant-derived natural products have been extensively studied throughout history as potential sources of drugs for the treatment of various major ailments. So far, numerous dedicated efforts have been undertaken to endorse and verify the possible impacts of experimental research and clinical use of natural products [17]. In recent times, a significant quantity of natural substances including plants like *Zingiber officinale*, Syzygiumaromaticum, *Curcuma longa*, *Trigonella foenumgraecum*, *Piper nigrum*, *Coriandrum sativum*, *Ocimum sanctum*, *Pterocarpus santalinus* and natural compounds like, berberine, curcumin, and resveratrol have been documented to have anti-DN effects in preclinical investigations [18].

*Crateva religiosa* is a sacred traditional plant that has been worshiped for centuries. Traditionally, several tribal and rural Communities have utilized it to treat various conditions such as Kidney Stones, Hypertension, Diabetes, Respiratory diseases, Pain, Inflammation, and more. The plant’s traditional literature documents that both traditional and alternative medicinal practitioners utilize and prescribe formulations containing *C. religiosa* to treat various diseases [19,20]. CR is abundant in phytochemical constituents and volatile oils. These compounds are known for their potent antioxidant, anti-inflammatory, immunomodulator and anti-apoptotic properties. Oxidative stress and apoptosis play significant roles in the pathogenesis of DN [21,22]. CR is vital for reducing oxidative stress and safeguarding podocytes—critical cells involved in the progression of DN [23,24]. Dyslipidemia, an important risk factor for cardiovascular disease, is frequently implicated in DN [25]. The extract of CR bark exhibited a promising hypolipidemic effect [26]. Similarly, inflammation in diabetic nephropathy leads to renal damage through the release of pro-inflammatory cytokines and activation of immune cells [27,28]. Tripathy et al reported that the alcoholic extract exhibits a more pronounced anti-inflammatory effect compared to the aqueous extract [29]. Traditionally, it has been used for immunity enhancement, restless leg syndrome, weight loss, and as an astringent and cholagogue. Additionally, it supports bone strength, improves urination and excretion, reduces heart disease risk, and promotes proper growth. It also functions as an antiemetic, an antidote for snakebites, aids digestion, increases appetite and biliary secretion, and acts as a laxative. Furthermore, it helps with convulsions, swelling, burning sensations in the feet, vesicant effects, and neurological pain relief [30–32].

Network pharmacology is a bioinformatics tool that is used to assess the various potential targets, roles, interactions, and pathways of bioactive components in disease treatment. It is considered a new approach to the discovery of drugs [33]. The drug-target network plays a crucial role in network pharmacology since it provides insights into the actions of drugs. The illness target facilitates the manufacturing of aids drugs, the discovery of drugs, and the identification of biomarkers by offering a thorough depiction of drug pathways in the treatment of the disease [34]. The concept of many targets for one drug originated from network pharmacology, which signifies a shift from the traditional approach of one drug targeting one specific gene to the idea of a drug having the ability to target numerous genes simultaneously [35]. The disease-associated genes like PI3K-AKT, PPARG and PTGS2 have biological importance in diabetic nephropathy. AKT is an important component of the PI3K-AKT signaling pathway. This pathway is involved in kidney damage by oxidative stress, cell apoptosis, inflammation, EMT, and autophagy. So, inhibition of the AKT1 gene could prove beneficial in mitigating kidney damage [36,37].AKT1-mediated mitochondrial dysfunction may play an important role in nephropathy and consequent renal failure [38]. AKT involvement in diabetic nephropathy and kidney damage is highlighted by many studies. The role of AKT in diabetic nephropathy and kidney impairment has been extensively investigated in various studies [39–43]. The ligand-activated nuclear transcription factors known as peroxisome proliferator-activated receptors (PPARG) are crucial for maintaining glucose and lipid homeostasis. PPAR agonists improve dyslipidemia and insulin resistance. They are nephroprotective by inhibiting oxidative stress, inflammation, activation of the RAS system, and lipotoxicity [44–47]. PTGS2, the gene that codes for the cyclooxygenase-2 (COX-2) enzyme, is involved in diabetic nephropathy by causing inflammation in the kidney through the synthesis of prostaglandins, especially prostaglandin E2 (PGE2), which can result in increased vascular permeability, proliferation of mesangial cells, and eventually renal damage [48–50]. Network Pharmacology contributes to 40% of active constituent discoveries and has a high rate of clinical development. Therefore, drug discovery is attracting growing attention [51].

Based on the above facts and their applicability to the molecular-based pharmacological assessment of phytochemicals, the present study was associated with exploring the molecular mechanistic role of *C. religiosa* phytochemicals in the treatment of diabetes and diabetic nephropathy (DN) and generating scientific evidence which would lay a stable foundation for further research on exploring its pharmacological mechanisms. Furthermore, this study would also narrow the activity-guided screening of phytoconstituents in laboratory animals and reduce the cost associated with their use, care and handling.

## 2. Materials and methods

### 2.1. *Crateva religiosa* (CR) bioactive components identification

The IMPPAT 2.0 database (https://cb.imsc.res.in/imppat/) was utilized to detect the bioactive constituents in CR. The compounds were chosen based on their oral bioavailability (OB) threshold of ≥30%, which was determined using the ADMETlab platform [52]. Additionally, their drug-likeness (DL) was assessed according to Lipinski’s rule of five. The oral bioavailability (OB) threshold indicates the fraction of a drug that enters the bloodstream after oral administration, taking into account its appropriate pharmacokinetic and physicochemical characteristics. Whereas DL is defined as the set of chemical and physical characteristics that establish a compound’s potential to be a safe and effective medicinal agent [53]. Lipinski’s guidelines offer a standardized approach to evaluate the drug-like characteristics of substances. These computational approaches have limitation to capture few biological complexities within therapeutic processes including protein binding and disease-specific microenvironmental effects. However, Network pharmacology and PPI-based analyses improve the biological significance of selected targets as they establish robust platform for future research.

### 2.2. ADME and bioavailability radar

SwissADME [54] and ADMETlab [55] were used to assess the ligands’ absorption, distribution, metabolism and elimination (ADME) characteristics. ADMETlab was utilized to determine important pharmacokinetic characteristics, such as Caco-2 permeability, topological polar surface area (TPSA), hydrogen bond acceptors (nHA) and donors (nHD), distribution (LogD), lipophilicity (LogP), solubility (LogS), and nHA. Using SwissADME, the impact on cytochrome P450 (CYP450) enzymes was also evaluated. The radar graph produced by ADMETlab offered a visual representation of these parameters, indicating that the ligands satisfy the requirements for drug-like properties.

### 2.3. Determining potential targets for CR bioactive components

In order to determine possible targets for the active components in CR, the Swiss Target Prediction web server (https://www.swisstargetprediction.ch/) was used. This platform utilizes a comparison of bioactive compounds to known ligands, using both two- and three-dimensional similarities for the identification of targets [56]. The SMILES of the bioactive components were submitted to the server, with “*Homo sapiens*” specified as the selected species. The official names of drug targets were obtained using the UniProtKB search tool in the UniProt database (https://www.uniprot.org/), and the prediction results were sorted on the basis of probability value (probability  >  0). Cytoscape 3.9.1 was used to create a “drugs-ingredients-targets” network map.

### 2.4. Acquisition of diabetic nephropathy disease targets

To find genes associated with diabetic nephropathy, search terms “Diabetic nephropathy” or “Diabetic kidney disease” were used in the DisGeNET database (https://www.disgenet.org/home/) and the GeneCards database (https://www.genecards.org/). It was believed that the disease target for DN was the intersection of these genes.

### 2.5. Constructing venn diagrams and PPI networks for data integration

Venn tool (https://bioinformatics.psb.ugent.be/webtools/Venn/) was used, to better understand the connections between diabetic nephropathy targets and the possible targets of CR. The shared target interactions were visualized by building a Protein-Protein Interaction (PPI) network with the STRING database (https://cn.string-db.org/) [57]. The list of 51 genes obtained after the intersection of the disease target and CR target was input into the STRING database (https://string-db.org/), with the species set as “homo sapiens”, the minimum required interaction score set as ≥0.4 [58], free targets hidden, and the rest of the parameters remained the default. In STRING, each protein–protein interaction is annotated with one or more ‘scores’. Importantly, these scores do not indicate the strength or the specificity of the interaction. Instead, they are indicators of confidence, i.e., how likely STRING judges an interaction to be true, given the available evidence. All scores rank from 0 to 1, with 1 being the highest possible confidence.

### 2.6. Hub gene identification

Once the PPI network was established, hub gene networks were constructed using Cytoscape 3.9.1 [59]. The hub genes were identified by using the “Degree” method. This method involves the analysis of weighted connections between nodes and the network topology with the help of cytoHubba plug-in in Cytoscape. Moreover, nodes with high topological relevance in the PPI network were selected using the Cytoscape plugin CytoNCA. The selected metrics for this investigation comprised Closeness centrality (CC), Degree centrality (DC), and betweenness centrality (BC). Nodes that had values higher than the median for all three topological attributes were chosen to construct a sub-network. Afterward, a second screening process was carried out to ultimately identify the main targets.

### 2.7. GO and KEGG analysis

The intersecting therapeutic targets were analyzed for functional enrichment using the gProfiler tool [60], GO enrichment analysis provided information on the biological mechanisms associated with the therapeutic targets, including biological processes (BP), molecular functions (MF), and cellular components (CC). KEGG enrichment analysis provided functional pathway annotations for these targets. The GO terms were analyzed and visualized using gProfiler, and the KEGG pathway enrichment analysis was also performed and visualized using the same tool. This comprehensive approach provided detailed insights into the biological roles and pathways relevant to the therapeutic targets.

### 2.8. Molecular docking verification of the phytoconstituents-target interaction

To validate compound-protein target interaction, the Molecular Operating Environment (MOE) (v2015.10) was used in this investigation [61]. The protein targets’ crystal structures were obtained using the RCSB PDB database (http://www.rcsb.org/). In order to build the receptor protein grid, which was connected to the procedures of protonation, elimination of molecules of water and repeated structure, structure preparation, and energy minimization, the imported crystal structure was imported into MOE. The binding location with ligands was chosen using the receptor’s grid construction method. In order to acquire the graphical results of molecular docking, the 2-dimensional chemical structure was downloaded from PubChem https://pubchem.ncbi.nlm.nih.gov/, loaded into MOE, and docked with protein construct. The Triangle Matcher placement method and the London dG scoring function were used in docking simulations to rank ligand poses. The main results were the identification of docking sites and the determination of free binding energies. The results also highlighted the preferred interaction sites and binding affinities of the ligands with the target protein, offering important insights into the molecular mechanisms of binding and possible therapeutic implications.

## 3. Results

### 3.1. *Crateva religiosa* (CR) bioactive components identification

From the IMPPAT database, 68 bioactive components were obtained (S4). Five phytochemicals met the criteria of ≥30% for bioavailability and did not violate any drug-likeness rules, including Lipinski, Ghose, and Veber (0 violation of three rules), as indicated in Table 1.

**Table 1:** Bioavailability score and drug-likeness characteristics of Crateva phytoconstituents.

Molecules	Common Name	SMILES	Chemical formulas	F (30%)
CR-C1	Dillapiole	C = CCc1cc2c(c(OC)c1OC)OCO2	C_12_H_14_O_4_	0.016
CR-C2	β-Ionone	CC(=O)C = CC1 = C(C)CCCC1(C)C	C_13_H_20_O	0.005
CR-C3	10-epi-γ-Eudesmol	CC1 = C2CC(C(C)(C)O)CCC2(C)CCC1	C_15_H_26_O	0.012
CR-C4	Cis-linalool oxide	C = C[C@@]1(C)CC[C@@H](C(C)(C)O)O1	C_10_H_18_O_2_	0.005
CR-C5	Trans-Linalool oxide	C = C[C@]1(C)CC[C@@H](C(C)(C)O)O1	C_10_H_18_O_2_	0.372
CR-C6	Nerolidol	C = CC(C)(O)CC/C = C(\C)CCC = C(C)C	C_15_H_26_O	0.01

### 3.2. Impact on pharmacokinetics, drug-likeness and CYP 450 enzymes

**Fig 1:**
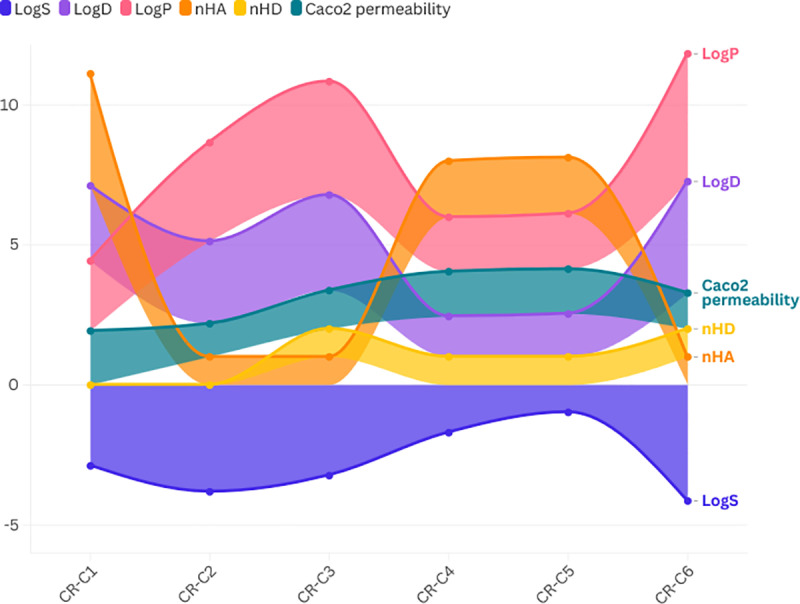
Absorption, distribution and bioavailability parameters of *Crateva religiosa.* Log S (Solubility): optimum range (‐4 to 0.5); Log P (Partition Coefficient): optimum range (0–3); Log D (Distribution Coefficient): optimum range (1–3); nHA (Number of Hydrogen Bond Acceptors): optimum range (0–12); nHD (Number of Hydrogen Bond Donors): optimum range (0–7); Caco-2 Permeability: optimum range (0.9–2).

The absorption properties of the six bioactive components found in *Crateva religiosa* are shown in Fig 1 (S1 Table). CR-C1 demonstrated the highest human intestinal absorption (96.80%) among the analyzed bioactive components. The remaining components showed decreasing HIA values in the following order: CR-C2 (95.79%), CR-C4, and CR-C5 (both at 94.77%), CR-C3 (93.67%), and CR-C6 (93.42%).

**Fig 2:**
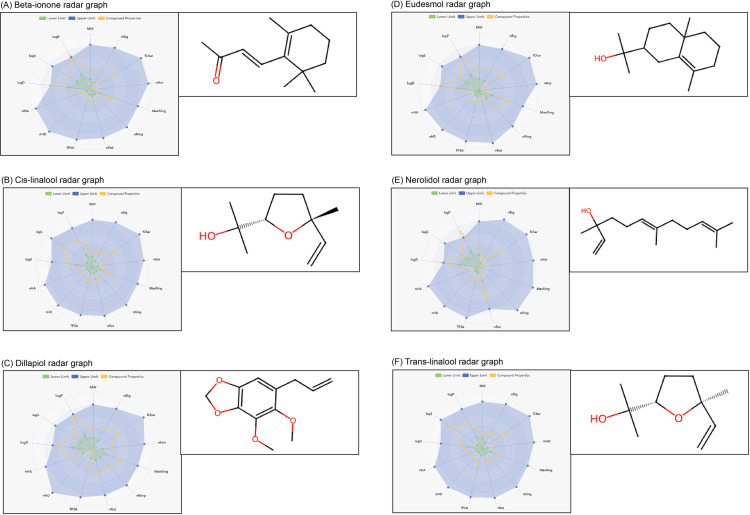
A radar graph displaying the predicted values of different physicochemical and molecular properties of *C. religiosa* phytoconstituents. MW stands for molecular weight. A number of rigid bonds (nRig) and formal charge (fChar) are important factors to consider. The value of nHet represents the number of heteroatoms present. MaxRing represents the number of atoms in the largest ring, while nRing indicates the total number of rings. The number of rotatable bonds is calculated as nRot. TPSA refers to the topological polar surface area. Number of hydrogen bond donors (nHD) and number of hydrogen bond acceptors (nHA) LogP is the logarithm of the octanol/water partition coefficient. LogS represent the logarithm of the solubility in water, while LogD represents the logarithm at a pH of 7.4, which is similar to the pH found in the human body.

The radar graph analysis revealed that the six selected bioactive components’ physical, chemical, and molecular characteristics were all within the ideal range (Fig 2). This suggests that these compounds possess favorable pharmacokinetic properties, including good solubility, distribution, and membrane permeability, which are crucial for their potential therapeutic efficacy in the management of diabetic nephropathy.

### 3.3. Metabolic profile of Crateva’s compound

**Fig 3:**
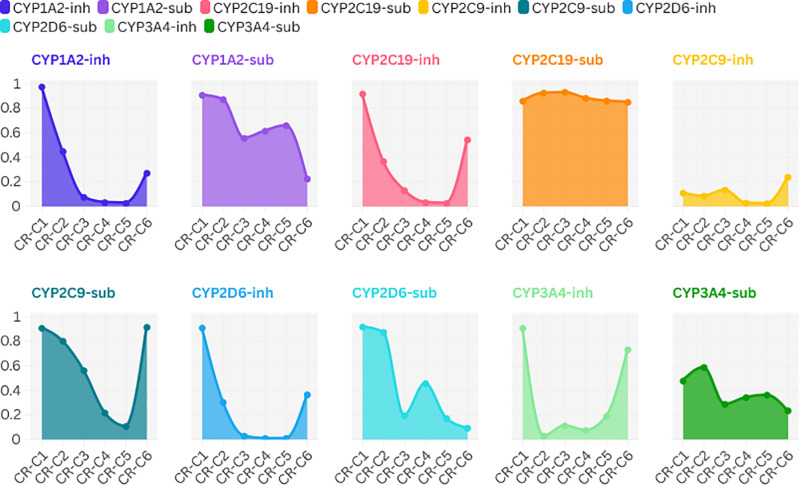
Summary of the effect of *C. religiosa* phytoconstituents on CYP450 enzyme. The ADMETlab tool predicts CYP inhibition (inh) and substrate (sub) activity values for 6 molecules using their SMILES strings. CYP enzymes (cytochrome P450 family) are responsible for the metabolism of a large number of compounds, and they are essential in determining whether a compound inhibits or is metabolized by CYP enzymes.

Six compounds were assessed by ADMETlab in terms of metabolism, with special attention to the interaction with the main CYP450 enzymes, namely CYP1A2, CYP2C19, CYP2C9, CYP2D6, and CYP3A4 (Fig 3). The enzymes are essential for phase I drug metabolism (which is most of the liver’s oxidative processes). These results provide insight into whether the chemicals act as inhibitors (acting on the activity of the enzymes) or substrates (metabolized by the enzymes). Values close to 1 indicate higher interaction probabilities and lower values indicate less influence (S2 Table).

Overall, the compounds demonstrated reasonable metabolic stability with a balanced interaction profile across the studied CYP enzymes. CR-C1 exhibits moderate potential for CYP enzyme interaction as a substrate or inhibitor, though its metabolic impact remains within an acceptable range for further therapeutic consideration. Compounds CR-C3, CR-C4, and CR-C5 are identified as CYP2C19 substrates with minimal inhibitory effects, suggesting the presence of efficient metabolic pathways without major enzymatic disruption. Similarly, CR-C6 shows mild interactions with CYP2C9 and CYP3A4, aligning with its predictable metabolic profile. While these interactions indicate the potential for CYP-mediated metabolism, no significant concerns regarding enzyme inhibition or metabolic interference were observed. These findings support the physiological acceptability of the compounds and justify their further evaluation in experimental settings to confirm their metabolic fate and pharmacokinetic viability.

### 3.4. Target identification and interaction network analysis of CR bioactive components

**Fig 4:**
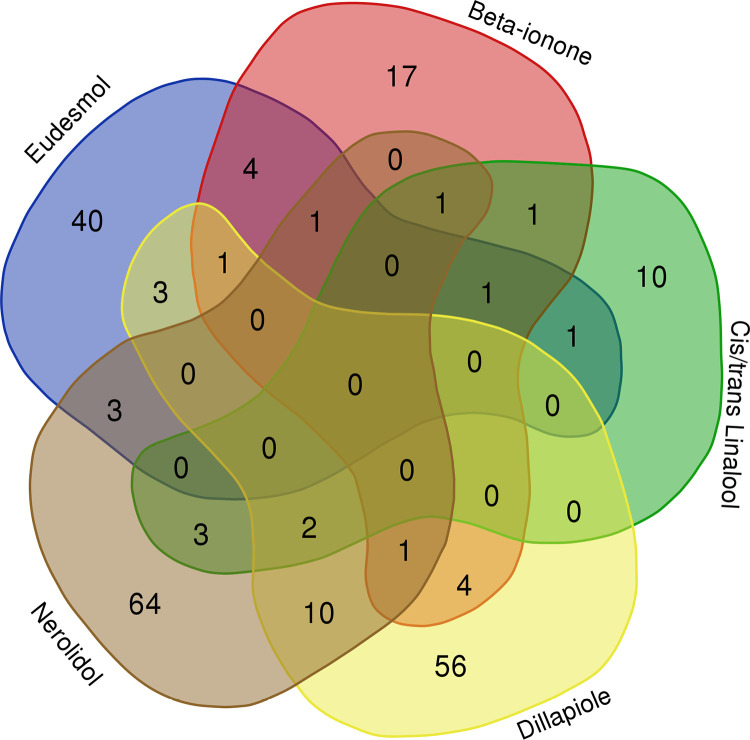
Predicted target count for five bioactive components using SwissTargetPrediction.

Using the PubChem database, SMILES of chosen bioactive components were searched, and the related targets were then obtained by importing the data into the SwissTarget Prediction platform.40 unique targets for CR-C3, 17 for CR-C2, 64 for CR-C6, and 10 for CR-C4/5 were predicted (Fig 4). Interaction Network was constructed between bioactive components of CR and predicted targets. This network had 239 nodes (6 CR bioactive components nodes and 233 common target nodes) and 311 edges. It was found that CR-C6 had the highest degree value (101) compared to other bioactive components. While the degree values of CR-C1, CR-C2, CR-C3, CR-C4 and CR-C5 were 85, 54, 32, 20 and 21 respectively. According to this finding, Crateva mostly used these compounds to play anti-DN roles.

### 3.5. Acquisition of diabetic nephropathy disease targets

The 4050 DN-related targets were acquired from GeneCards and 1189 from DisGeNET. After removing duplicates, 2658 targets were identified.

### 3.6. Constructing Venn diagrams and PPI networks for data integration

**Fig 5:**
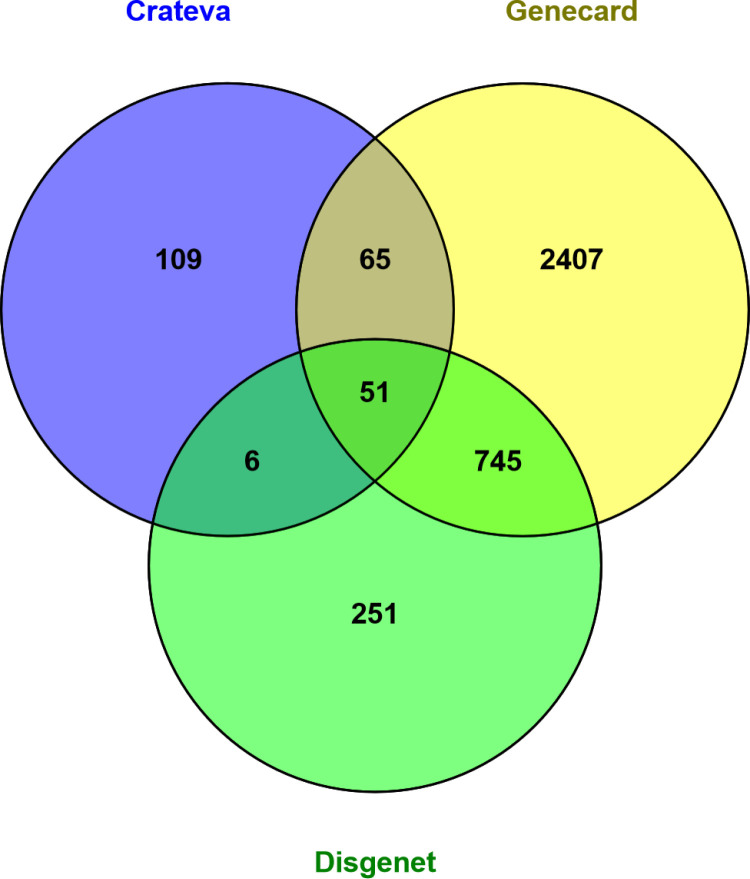
Common targets between Crateva, GeneCard and DisGenet.

**Fig 6:**
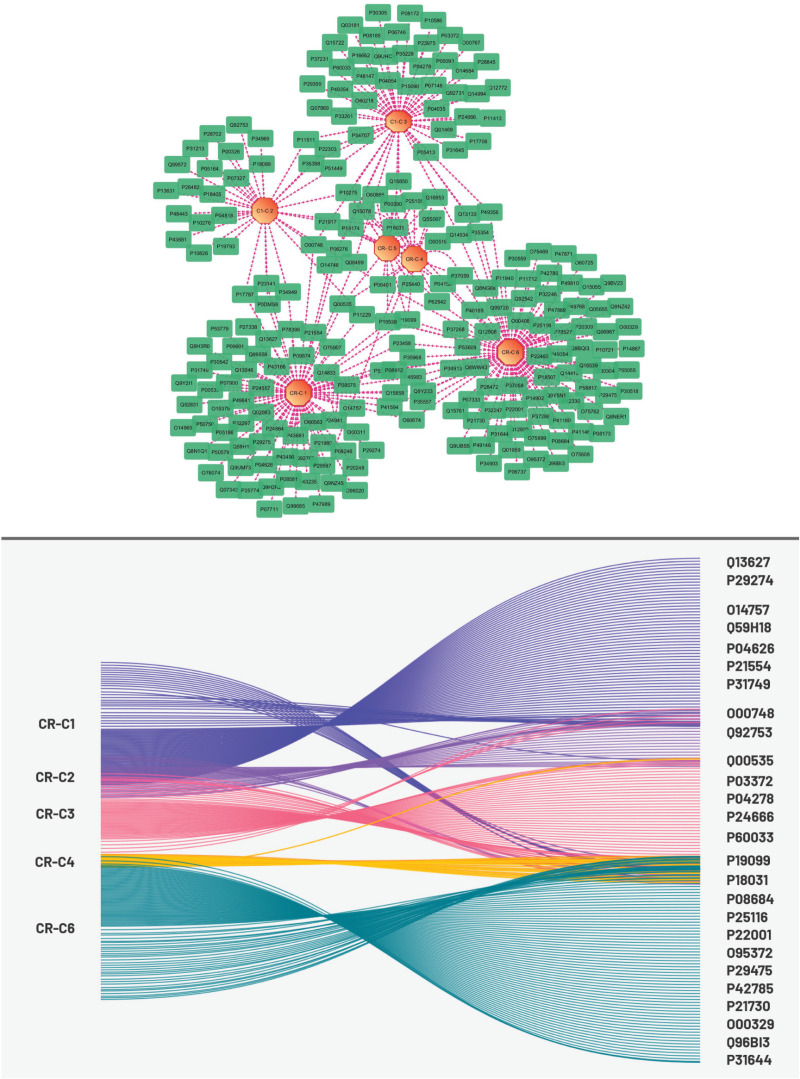
Network visualization of interactions between Crateva’s phytochemicals, disease targets, and phytochemical targets. The figure illustrates the interaction network of key plant phytochemicals (CR-C1, CR-C2, CR-C3, CR-C4, CR-C5 and CR-C6) with their respective disease targets and phytochemical.

51 common targets between CR bioactive components, DisGenet and GeneCards were obtained by using the Venn diagram tool (Fig 5). By importing these 51 common targets into the STRING database (S3 Table), a PPI network was created to examine the mechanism behind the therapeutic actions of CR against DN. The network had 207 edges and 42 nodes in total (Fig 6). The prioritization of genes was based on three key topological parameters: Degree Centrality (DC), Closeness Centrality (CC), and Betweenness Centrality (BC). Degree Centrality reflects the number of direct connections a gene has with others in the network, providing insight into its prominence within the system. Closeness Centrality measures the average shortest path that a gene shares with all other genes, indicating its accessibility within the network. Betweenness Centrality denotes the frequency with which a gene lies on the shortest path between pairs of other genes, highlighting its potential influence in mediating interactions [62].

### 3.6. Hub Genes identification

**Fig 7:**
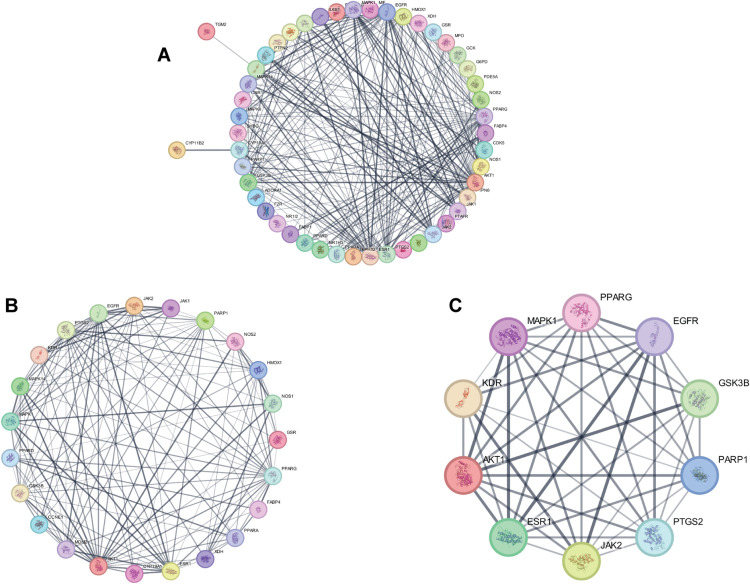
Protein-protein interaction for identification of candidate Targets. (A), Network of 51 common genes between CR bioactive components and disease genes. (B) sub-network of genes based on the median value of closeness, betweenness and degree (C). Network of top 10 hub genes.

For the identification of Hub Genes, three topological characteristics of the 42 nodes were computed using a cytoHubba and cytoCNA plugin of Cytoscape. These nodes’ respective median CC, DC, and BC values were 0.516, 8, and 0.003 respectively. Nodes having values of all three topological characteristics above the median were chosen to construct a sub-network, as seen in Fig 7.

To ultimately acquire the central targets, additional selection was made in the sub-network (Fig. 6C). Ultimately, ten genes AKT1, PPARG, PTGS2, EGFR, ESR1, JAK2, MAPK1, PARP1, GSK3B, PPARA were found to be the primary therapeutic targets of Crateva’s activities against DN.

### 3.7. Effect on GO and KEGG analysis

**Fig 8:**
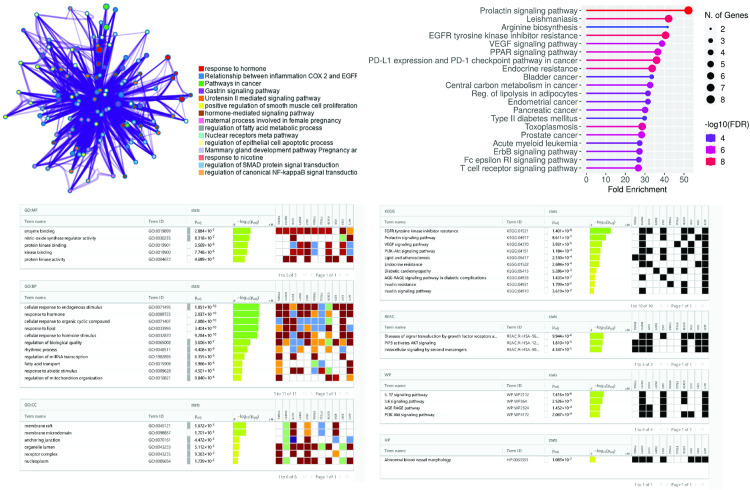
GO and KEGG analysis. The figure illustrates the enriched pathways and gene ontology terms identified through gProfiler analysis. KEGG pathway enrichment reveals significant pathways such as the Prolactin signaling pathway, VEGF signaling pathway, and Type II diabetes mellitus pathway. The Gene Ontology (GO) analysis encompasses three categories: Biological Process (GO BP), Molecular Function (GO MF), and Cellular Component (GO CC). GO BP terms include cellular response to endogenous stimulus, response to hormones, and cellular response to organic cyclic compounds. GO MF terms encompass enzyme binding and nitric oxide synthase regulatory activity. GO CC terms highlight membrane raft and membrane microdomain localization. These analyses provide a detailed functional annotation of the target proteins, elucidating their roles in various biological processes and molecular activities.

The 51 overlapping targets were queried into g: profiler webserver for GO and KEGG pathway analyses in order to identify the underlying mechanisms of CR therapeutic effect on DN. Significant correlations with 666 biological processes (BP) were found by the GO functional enrichment analysis. The processes that ranked highest by P-value included cellular response to endogenous stimulus, response to hormones, cellular response to organic cyclic compound and response to lipid as shown in Fig 8. There were 25 entities associated with cellular components (CC), some of which were prominent structures including membrane rafts, membrane microdomain, and anchoring junctions. 56 molecular functions (MF) were identified; the most significant ones were Nitric oxide synthase regulator activity, enzyme binding, and protein kinase binding.

The KEGG pathways encompassed several aspects of human diseases, including signaling pathways and pathophysiological mechanisms. The most common signaling pathways were prolactin signaling pathway, PPAR signaling pathway, diabetic cardiomyopathy, Insulin resistance, VEGF signaling pathway, FoxO signaling pathway, lipid and atherosclerosis, nuclear receptors, Urotensin II-mediated signaling pathway, AGE-RAGE pathway, IL 26 signaling pathways, PPAR signaling pathway, Oxidative stress response and IL-6 signaling pathway. These findings indicated that Crateva may play a role in the treatment of DN by regulating the key targets in these signaling pathways, and most therapeutic targets participate in multiple signaling pathways.

### 3.8. Molecular docking

**Fig 9:**
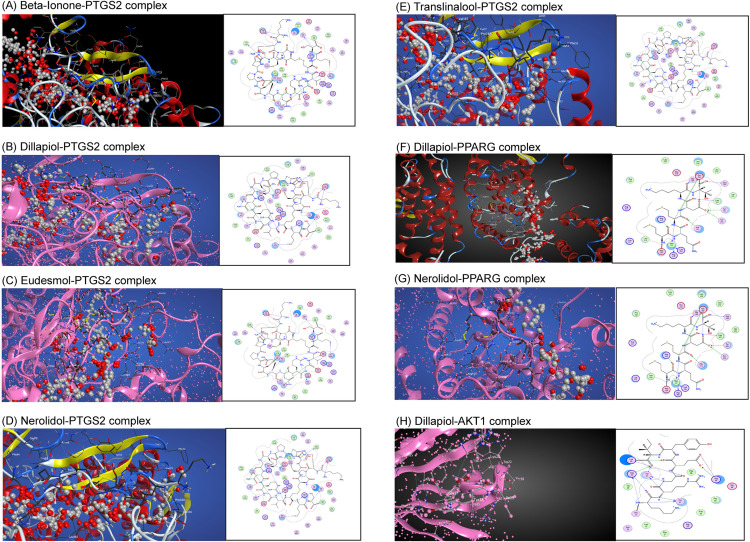
Virtual docking of active phytoconstituents of *C.religiosa* against Top 3 therapeutic targets. (A) Beta-Ionone-PTGS2 complex, (B) Dillapiol-PTGS2 complex, (C) Eudesmol-PTGS2 complex, (D) Nerolidol-PTGS2 complex, (E) Translinalool-PTGS2 complex, (F) Dillapiol-PPARG complex, (G) Nerolidol-PPARG complex, (H) Dillapiol-AKT1 complex.

The chosen targets, namely AKT1, PPARG, and PTGS2, had a crucial function in the CR-DN network for therapeutic targets and hold the highest degree rank. The top five compounds in the CR-DN network, ranked by the count of linked target genes, were CR-C6, CR-C1, CR-C2, CR-C3, and CR-C4/5. Three target genes and five chemicals were loaded into MOE for molecular docking verification. The docking scores are displayed in Table 2. Fig 9 illustrates the action modes of CR-C6, CR-C1, CR-C2, and CR-C3 in relation to the top three genes (AKT1, PPARG, and PTGS2). The binding energies ranged from ‐5.097 to ‐6.924 kcal/mol, indicating stable and favorable docking poses. With a binding energy of ‐6.924 kcal/mol, CR-C6 showed the highest binding affinity of all the bioactive components with PTGS2, suggesting that it could be a lead molecule for future therapeutic development. CR-C1 demonstrated robust interactions with PPARG and AKT1. The stability of ligand–protein complexes can be measured through refinement energy (E_refine) after conducting energy minimization. A lower E-refine value demonstrates better stabilization and stronger binding adaptability within the active site of target proteins. The docking results showed that CR-C1 achieved the most stable interaction with AKT1 with an E_refine value of ‐47.158. CR-C6 and CR-C2 also displayed a favorable E-refine value of 32.674 and 24.941 respectively against PTGS2 protein, which strengthens its potential to bind effectively. These findings open the door for further experimental validation and optimization by offering insightful information about the interaction processes and possible therapeutic uses of the bioactive components.

**Table 2:** Binding energy of bioactive components against therapeutic targets.

Targets	Ligands	Binding Energy Kcal/mol	E_refine values	Active site Residue of Target
PTGS2	CR-C3 CR-C6 CR-C5 CR-C2 CR-C1	-5.5367 -6.9248 -5.0188 -5.8979 -5.9542	-16.626 -32.674 -19.743 -24.941 -28.48	(LYS33 ASN34 PRO35 CYS36 CYS37 SER38 HIS39 PRO40 CYS41 GLN42 ASN43 ARG44 GLY45 VAL46 CYS47 MET48 SER49 PHE52 THR60 ARG61 THR62 GLY63 PHE64 LEU75 THR76 LYS79 LEU80 LEU82 LYS83 PRO84 THR85 PRO86 ASN87 VAL89 HIS90 LEU93 TYR115 VAL116 THR118 SER119 ARG120 SER121 HIS122 LEU123 ILE124 ASP125 SER126 PRO127 THR129 TYR130 ALA132 ASP133 TYR134 GLY135 TYR136 L... RG513 GLU520 VAL523 GLU524 LEU531 LYS532)2:(GLU322 TRP323 GLY324 ASP325 GLU326 GLN327 GLN330 THR331 LEU334 LEU366 PHE367 LYS369 GLN370 PHE371 TYR373 SER541 PRO542 ALA543 LYS546 SER548 THR549 PHE550 GLY551)
PPARG	CR-C6 CR-C1	-6.228 -5.0975	-24.5 -18.9204	(LEU240 ALA241 VAL242 GLU243 PRO244 LYS245 THR246 THR248 LYS274 GLN275 PHE277 THR278 VAL280 GLU281 LYS284 ARG285 ALA327)3:(ASN227 GLU228 MET230 PRO231 VAL232 GLU233 LEU236 HIS315 ILE318 MET357 ARG358 MET360 GLN361 MET362 ASP363 LYS364 THR365 SER399 ALA402 TYR403 HIS406 LYS407 TYR408 ARG414)5:(ARG635 LEU636 GLU639)
AKT1	CR-C1	-5.3445	-47.158	(LYS14 ARG15 GLY16 GLU17 TYR18 ILE19 ARG23 ARG25 LEU52 ASN53 ASN54 PHE55 GLN79 ARG86)

## 4. Discussion

Diabetic nephropathy (DN) is a severe consequence of diabetes that frequently leads to end-stage renal disease. Novel therapeutic approaches are needed to address this problem, and network pharmacology presents a viable strategy by combining systems biology and bioinformatics to comprehend intricate drug-disease interactions. This study examined the therapeutic properties of the *Crateva religiosa* (CR) plant in DN. Five important phytoconstituents: CR-C1, CR-C2, CR-C3, CR-C4/5 and CR-C6 were identified. The interactions between these bioactive components and significant genes associated with DN, including AKT1, PPARG, and PTGS2, were examined.

The bioactive substance CR-C1 has been studied for possible effects on diabetic nephropathy. CR-C1 is an ingredient in the Sishenwan formula, which has been shown in a study to modulate AGE-RAGE, IL-17, and TNF signaling pathways to produce therapeutic benefits on diabetic nephropathy. The study found important targets, including TNF, CREB1, and PTGS2, indicating that CR-C1 may be important in lowering oxidative stress and inflammation in diabetic nephropathy [63]. Similarly, Mahnashi et al; 2022 reported the strong blood glucose-lowering effects of CR-C1 in experimental animals [64].

Jiang and Zhang (2022) reported that CR-C6 significantly enhanced the activities of carbohydrate metabolic enzymes and augmented glycogen storage in the liver of diabetic rats. In diabetic rats, CR-C6 treatment increased serum high-density lipoprotein-C levels, while a marked reduction was observed in serum triglycerides, total cholesterol (C), low-density lipoprotein-C, and very low-density lipoprotein-C. However, further experimental validation is necessary to confirm its direct effects on oxidative stress markers [65].A study conducted by Silvy Mathew and S. John Britto demonstrated that Nerolidol has anti-diabetic effect [66]. The anti-DN action of CR-C6 is attributed to its anti-inflammatory, anti-oxidant, and anti-diabetic properties [67].

Deepa and Anuradha (2013), in an experiment with diabetic rats, reported that treatment with linalool reinstated the activity of enzymes involved in glucose metabolism, increased collagen levels, and restored the expression of GLUT-1. Linalool exerted a protective effect on the kidney by reducing oxidative stress and inflammation through downregulation of the expression of TGF-β1 and NF-kB. Linalool attenuated ultrastructural alterations observed in diabetic mice, including basement membrane thickening, reduction in podocyte number, and loss of filtration barrier integrity [68]. By lowering kidney inflammation and preventing more damage, linalool oxide’s regulation of inflammatory pathways can help maintain renal function.

One important phytochemical that shows great therapeutic promise in the management of diabetic nephropathy is CR-C2. Its documented effects include a reduction in inflammation and oxidative stress, that accelerate the development of diabetic nephropathy. Research indicates that by focusing on pathways connected to inflammation and cellular stress responses, CR-C2 may be able to reduce kidney damage [69]. The release of NO, PGE2, and TNF-α is markedly inhibited by CR-C2. CR-C2 also suppresses the expression of TNF-α protein, cyclooxygenase-2 (COX-2), and inducible NO production (iNOS). β-Ionone significantly suppresses the phosphorylation of mitogen-activated protein kinases (MAPKs), such as p38, ERK1/2, and JNK, which are intimately associated with controlling the release of pro-inflammatory mediators[70].These pro-inflammatory mediators play an important role in the pathogenesis of DN.

The therapeutic target-PPI network identified AKT1, PPARG, PTGS2, EGFR, ESR1, JAK2, MAPK1, PARP1, GSK3B, PPARA as the major targets of *C. religiosa*.

AKT1 is a pivotal signaling protein that plays a critical role in cell survival, growth, and metabolism. The AKT1 signaling pathway plays a crucial role in safeguarding renal cells from apoptosis and preserving podocyte function in diabetic nephropathy. Studies have demonstrated that mitochondrial AKT1 plays a crucial role in the mechanisms of kidney injury, specifically in the context of ischemia-reperfusion injury. Reduced AKT1 signaling in the presence of high blood sugar levels causes an elevation in oxidative stress and programmed cell death in kidney cells, worsening the extent of kidney injury [38,71].

PPARγ has significant functions in both normal kidney function and abnormal kidney conditions. PPARγ agonists have a beneficial impact on several kidney illnesses, such as diabetic nephropathy (DN), polycystic kidney disorders, IgA nephropathy, chemotherapy-associated kidney damage, ischemic renal injury, and age-related kidney diseases. These advantages are achieved by actions in both the whole body and the kidneys [72]. It has been demonstrated that PPARG agonists, such as thiazolidinediones, enhance renal outcomes in diabetic nephropathy by increasing insulin sensitivity, lowering inflammation, and lowering albuminuria [73]. Pro12Ala polymorphism in the PPARG gene has also been linked to decreased risk and severity of diabetic nephropathy, emphasizing the role of genetics in the development of the illness [74].

According to recent research, prostaglandin-endoperoxide synthase-2 (PTGS2), sometimes referred to as cyclo-oxygenase 2, may be involved in the etiology of type 2 diabetes mellitus (T2DM). PTGS2 produces prostaglandins, which have a detrimental effect on glucose-stimulated insulin release. It also acts as a mediator of the inflammatory response, that is linked to reduced insulin sensitivity [49]. COX-2, is involved in mediating the inflammatory response. Its overexpression causes glomerular hyperfiltration, fibrosis, and elevated inflammation in the kidneys of diabetes individuals [48]. Because proteinuria and renal inflammation have been demonstrated to be reduced by selective COX-2 inhibitors, PTGS2 may be a viable therapeutic target for the treatment of diabetic nephropathy.

EGFR expression and activation are increased in experimental models of DKD as well as in cultured renal cells subject to high glucose [75,76]. A recent study has discovered a single nucleotide polymorphism (SNP) in an enhancer region within the EGFR gene that is linked to an increase in the expression of EGFR in individuals with type 2 diabetes [77]. Furthermore, other EGFR ligands, such as TGF-α, HB-EGF, and amphiregulin, have been observed to elevate in experimental models of DN [75,78].

The GO analysis revealed that there were significant relationships between the treatment targets and BP terms (cellular response to endogenous treatment, response to hormone, cellular response to organic cyclic compound, response to lipid) CC terms (membrane raft, membrane microdomain), and MF terms(enzyme binding, nitric oxide synthase regulatory activity, protein Kinase binding). Thus, the previously identified pathways may be used by Crateva for the treatment of DN. For example, blocking endothelial NO increases microvascular disease, which is known to impede renal autoregulation, in animal models of Chronic Kidney disease (CKD) and arteriosclerosis. Research has demonstrated that endothelial dysfunction can also result in an uncoupling of the VEGF-NO axis, which amplifies the proliferative and proinflammatory effects of VEGF [79,80]. NOS may contribute to the development of chronic diabetic complications via other pathways, including UCP2. Numerous tissues express UCP2, which is involved in fatty acid metabolism, beta cell regulation of insulin production, and protection against oxidative stress [81]. So Nitric oxide synthase gene may be a [82] good candidate for the treatment of DN.

Based on the KEGG terms, the therapeutic targets of *C. religiosa* against DN are primarily linked to the PI3K-Akt signaling pathway, Insulin resistance, insulin signaling pathways, AGE-RAGE signaling pathway in diabetic complications, and IL17 signaling pathway. Podocytes are responsible for maintaining the filtration barrier in the glomerulus, which is made up of the basement membrane and slit diaphragm. It has been observed that podocyte death is linked to an increase in macroalbuminuria in diabetic nephropathy.

A recent study examined Jiawei Shengjiangsan’s effects on diabetic nephropathy in mice, emphasizing the function of the PI3K/Akt/NF-κB signaling pathway. In addition to lowering renal inflammatory markers, the therapy also reduced blood urea nitrogen (BUN), urine albumin, and serum creatinine (SCr). Furthermore, there was a reduction in the amount of phosphorylated NF-κB p65 and an increase in phosphorylated PI3K and Akt levels, indicating the pathway’s crucial role in mitigating renal injury [83]. The role of PI3K-Akt signaling is essential for the survival of podocytes. Cell death may occur as a result of the pathway’s blockage because downstream targets may not be phosphorylated as much. Podocytes depend on PI3K-Akt signaling to sustain their survival and functionality. Any interruption in the pathway of podocytes can result in apoptosis, which can contribute to the development of renal disorders [84]. Huang et al. discovered that notoginsenoside R1 has the ability to activate the PI3K-Akt signaling pathway, resulting in anti-apoptotic and renal-protective actions in mice with diabetic nephropathy [85].

The AGE-RAGE signaling pathway plays a crucial role in the development of diabetic complications. The increase in advanced glycation end products (AGEs) levels and the elevation of the receptor for AGEs (RAGE) expression can worsen the course of diabetic nephropathy (DN) [86]. Prolonged activation of glycosylation of reducing sugars in the kidney leads to the gradual accumulation of AGEs, which increases the risk of extracellular matrix migration, renal tubular dysfunction, as well as glomerular proliferative lesion. In addition, advanced glycation end products (AGEs) have the ability to attach to receptors known as RAGE receptors, resulting in a persistent inflammatory response, oxidative stress, damage to kidney tissue, and a decline in kidney function [87]. According to Sharma et al., the interaction between AGE and RAGE accelerates the advancement of DN by causing the release of fibronectin, TGF-β, and inflammatory cytokines [88].

IL-17 has a role in fibrosis, immune dysregulation, and chronic inflammation linking it to the pathogenesis of DN. Activation of the IL-17 pathway in certain renal disorders can stimulate the production of inflammatory cytokines [89]. Inflammatory cytokines can induce glomerulosclerosis and renal tissue destruction in diabetic nephropathy through an inflammatory reaction [90,91]. IL-17 blocking agents could be useful in the treatment of DN [92]. IL-17 deficiency aggravates STZ-induced DN by inhibiting the autophagic response [93]. Several other studies also establish the potential role of IL-17 in diabetic nephropathy [94–100]. A novel therapeutic approach for DN exists through IL-17 modulation together with its downstream targets. The renoprotective effects of CR depend on its ability to lower IL-17 expression, which blocks mesangial cell PI3K-Akt activation and inhibits AGE-RAGE-induced ROS production. CR treatment disrupts the interconnected signaling network, helping prevent chronic inflammatory and fibrotic DN responses.Network Pharmacological analysis of CR discovered that the IL-6 signaling pathway (WP Term ID: WP364) also serves as a vital pathway in DN development because it highlights the importance of persistent inflammation in disease progression. The research by Al-Awaida et al. (2021) revealed that T2DM patients with higher IL-6 levels showed significant activation of Notch-2/Jagged-1 signaling which supports an inflammatory signaling mechanism in renal fibrosis development [101] ”.

Genetic variations in the IL-6 and Notch-2/Jagged-1 signaling pathways appear to modify the risk and severity of DN. Studies have shown that genetic mutations in the IL-6 gene result in elevated cytokine levels, which intensify renal inflammation and fibrosis[102,103].

Based on the multiple pathways discussed above, CR appears to modulate immune responses, oxidative stress, inflammation, and advanced glycation end products, which may contribute to renal protection and potentially slow the progression of diabetic nephropathy. Further studies will help to better understand its precise role in these processes.

## 5. Conclusion

CR-C1, CR-C2, CR-C3, CR-C4/5, and CR-C6 were essential phytoconstituents identified and recommended as anti-inflammatory, anti-oxidant, and immunomodulatory agents, targeting AKT1, PPARG, PTGS2, EGFR, ESR1, JAK2, MAPK1, PARP1, GSK3B, and PPARA. The PI3K-Akt signaling pathway, AGE-RAGE, and IL-17 pathways were promising signaling pathways in the therapy of DN with *C. religiosa.*

### 5.1. Limitations of the study

Network pharmacology benefits the drug discovery process, but this approach contains specific built-in constraints that should be recognized. It is limited in delivering comprehensive solutions regarding pharmacokinetic complexities, optimal dosing requirements, and experimental validations. Network-based analyses help identify important molecular interactions, but they fail to show the dynamic aspects and time-dependent patterns that diseases naturally follow. The drug discovery process heavily depends on network pharmacology as its initial vital step, even though it has certain shortcomings that need to be addressed.

## Supporting information

S1 Table Absorption, distribution, and bioavailability parameters of *Crateva religiosa.* (PDF)

S2 Table Summary of the effect of *C. religiosa* phytoconstituents on CYP450 enzyme. (PDF)

S3 Table 51 common Genes in “Crateva”, “Genecard” and “Disgenet”. (PDF)

S4 Table List of phytoconstituents of *C. religiosa.* (PDF)
