# Supplementary material for: Therapeutic role of Crateva religiosa in diabetic nephropathy: Insights into key signaling pathways
Source: PLoS One. 2025 May 28;20(5):e0324028. doi: 10.1371/journal.pone.0324028 (PMC12118869; doi:10.1371/journal.pone.0324028)
Supplement: S1 Table — (PDF) [file pone.0324028.s001.pdf]

S1 Table. Absorption, distribution and bioavailability parameters of Crateva religiosa.

Molecules	LogS	LogD	LogP	nHA	nHD	Caco2 permeability
CR-C1	-2.884	2.682	2.501	4	0	1.926
CR-C2	-3.816	2.938	3.535	1	0	1.186
CR-C3	-3.232	3.407	4.055	1	1	1.374
CR-C4	-1.701	1.446	1.947	2	1	1.598
CR-C5	-0.974	1.536	1.985	2	1	1.598
CR-C6	-4.139	3.981	4.561	1	1	1.283
Lower limit	-4	1	0	0	0	0.9
Upper Limit	0.5	3	3	12	7	2
