# Supplementary material for: Therapeutic role of Crateva religiosa in diabetic nephropathy: Insights into key signaling pathways
Source: PLoS One. 2025 May 28;20(5):e0324028. doi: 10.1371/journal.pone.0324028 (PMC12118869; doi:10.1371/journal.pone.0324028)
Supplement: S2 Table — (PDF) [file pone.0324028.s002.pdf]

S2 Table.Summary of the effect of *C. religiosa* phytoconstituents on CYP450 enzyme.

Molecules	CYP1A2-inh	CYP1A2-sub	CYP2C19-inh	CYP2C19-sub	CYP2C9-inh	CYP2C9-sub	CYP2D6-inh	CYP2D6-sub	CYP3A4-inh	CYP3A4-sub
CR-C1	0.972	0.905	0.915	0.857	0.107	0.906	0.908	0.915	0.906	0.476
CR-C2	0.446	0.869	0.365	0.922	0.083	0.798	0.301	0.871	0.027	0.586
CR-C3	0.073	0.554	0.128	0.928	0.132	0.56	0.026	0.193	0.11	0.285
CR-C4	0.031	0.613	0.031	0.879	0.026	0.215	0.007	0.454	0.073	0.341
CR-C5	0.025	0.657	0.024	0.858	0.022	0.105	0.007	0.17	0.189	0.36
CR-C6	0.269	0.222	0.542	0.848	0.237	0.913	0.363	0.09	0.729	0.232
