# Supplementary material for: Therapeutic role of Crateva religiosa in diabetic nephropathy: Insights into key signaling pathways
Source: PLoS One. 2025 May 28;20(5):e0324028. doi: 10.1371/journal.pone.0324028 (PMC12118869; doi:10.1371/journal.pone.0324028)
Supplement: S3 Table — (PDF) [file pone.0324028.s003.pdf]

S3 Table. 51 common Genes in "Crateva", "Genecard" and "Disgenet":		
SN	**Uni Prot ID**	
1	P19099	
2	P00390	
3	Q16853	
4	P25105	
5	O60885	
6	P14174	
7	Q00535	
8	P30542	
9	P00533	
10	P23458	
11	O60674	
12	O76074	
13	P35968	
14	P45983	
15	P35557	
16	P09874	
17	P09601	
18	P21554	
19	P21980	
20	P31749	
21	P24864	
22	P47989	
23	P49841	
24	Q13133	
25	P25116	
26	Q16539	
27	P32246	
28	Q00987	
29	P35354	
30	P30518	
31	P29475	
32	P40189	
33	O75469	
34	P34913	
35	P21730	
36	P11511	
37	P28482	
38	P05164	
39	Q99572	
40	P03372	
41	P04278	
42	P17706	
43	P04054	
44	P29350	
45	P15090	
46	P37231	
47	Q07869	
48	Q03181	
49	P07148	
50	P11413	
51	P35228	
