# Supplementary material for: Therapeutic role of Crateva religiosa in diabetic nephropathy: Insights into key signaling pathways
Source: PLoS One. 2025 May 28;20(5):e0324028. doi: 10.1371/journal.pone.0324028 (PMC12118869; doi:10.1371/journal.pone.0324028)
Supplement: S4 Table — (PDF) [file pone.0324028.s004.pdf]

**S4 Table. List of Phytoconstituents of *C. religiosa***				
**Indian medicinal plant**	**Plant part**	**IMPPAT Phytochemical identifier**	**Phytochemical name**	
Crateva religiosa	bark	IMPHY001856	Stachydrine	
Crateva religiosa	bark	IMPHY003681	Diosgenin	
Crateva religiosa	bark	IMPHY004101	1-S-[(1Z)-N-(sulfooxy)ethanimidoyl]-1-thio-beta-D-glucopyranose	
Crateva religiosa	bark	IMPHY007183	Spinasteryl acetate	
Crateva religiosa	bark	IMPHY007273	1-Hexacosanol	
Crateva religiosa	bark	IMPHY010181	(8E,22Z)-4-hydroxy-2-oxa-11,16,20-triazatricyclo[22.2.2.13,7]nonacosa-1(26),3,5,7(29),8,22,24,27-octaene-10,21-dione	
Crateva religiosa	bark	IMPHY011688	Friedelin	
Crateva religiosa	bark	IMPHY011990	(-)-Epiafzelechin	
Crateva religiosa	bark	IMPHY012003	Betulinic acid	
Crateva religiosa	bark	IMPHY012224	(-)-Catechin	
Crateva religiosa	bark	IMPHY012473	Lupeol	
Crateva religiosa	bark	IMPHY012936	Cadabicine diacetate	
Crateva religiosa	bark	IMPHY013714	Cadabicine methyl ether	
Crateva religiosa	bark	IMPHY014836	beta-Sitosterol	
Crateva religiosa	bark	IMPHY014991	Lupeol acetate	
Crateva religiosa	bark	IMPHY015071	stigmast-5-en-3beta-yl beta-D-glucopyranoside	
Crateva religiosa	bark	IMPHY015081	Taraxasterol	
Crateva religiosa	fruit	IMPHY002983	1-Hexadecanol	
Crateva religiosa	fruit	IMPHY004101	1-S-[(1Z)-N-(sulfooxy)ethanimidoyl]-1-thio-beta-D-glucopyranose	
Crateva religiosa	fruit	IMPHY006558	1-Triacontanol	
Crateva religiosa	fruit	IMPHY007273	1-Hexacosanol	
Crateva religiosa	fruit	IMPHY009413	Triacontane	
Crateva religiosa	fruit	IMPHY014836	beta-Sitosterol	
Crateva religiosa	leaf	IMPHY001856	Stachydrine	
Crateva religiosa	leaf	IMPHY004619	Quercetin	
Crateva religiosa	leaf	IMPHY012721	Isoquercitrin	
Crateva religiosa	leaf	IMPHY015047	Rutin	
Crateva religiosa	root	IMPHY003016	Lauric acid	
Crateva religiosa	root	IMPHY004100	Calendol	
Crateva religiosa	root	IMPHY004619	Quercetin	
Crateva religiosa	root	IMPHY004631	Stearic acid	
Crateva religiosa	root	IMPHY004705	Stigmast-5-en-3-yl acetate	
Crateva religiosa	root	IMPHY007183	Spinasteryl acetate	
Crateva religiosa	root	IMPHY011471	Lupenone	
Crateva religiosa	root	IMPHY011797	Oleic acid	
Crateva religiosa	root	IMPHY012473	Lupeol	
Crateva religiosa	root	IMPHY012723	Linolenic acid	
Crateva religiosa	root	IMPHY014836	beta-Sitosterol	
Crateva religiosa	root	IMPHY014838	Daucosterol	
Crateva religiosa	root	IMPHY014991	Lupeol acetate	
Crateva religiosa	root	IMPHY015047	Rutin	
Crateva religiosa	whole plant	IMPHY000308	Hexadecane	
Crateva religiosa	whole plant	IMPHY000795	Octanal	
Crateva religiosa	whole plant	IMPHY001144	Dillapiol	

Crateva religiosa	whole plant	IMPHY001915	Octadecane	
Crateva religiosa	whole plant	IMPHY003316	Pentadecanal	
Crateva religiosa	whole plant	IMPHY003525	Nonanal	
Crateva religiosa	whole plant	IMPHY003760	2-Nonenal	
Crateva religiosa	whole plant	IMPHY003915	2-Decenal	
Crateva religiosa	whole plant	IMPHY006337	1-Octen-3-one	
Crateva religiosa	whole plant	IMPHY006485	beta-Ionone	
Crateva religiosa	whole plant	IMPHY006668	Isophorone	
Crateva religiosa	whole plant	IMPHY006951	Eicosane	
Crateva religiosa	whole plant	IMPHY006970	Decanal	
Crateva religiosa	whole plant	IMPHY007076	Undecanal	
Crateva religiosa	whole plant	IMPHY007186	Heptanal	
Crateva religiosa	whole plant	IMPHY007204	Dodecanal	
Crateva religiosa	whole plant	IMPHY009368	Heptadecane	
Crateva religiosa	whole plant	IMPHY009375	Docosane	
Crateva religiosa	whole plant	IMPHY009382	Heneicosane	
Crateva religiosa	whole plant	IMPHY009389	Pentadecane	
Crateva religiosa	whole plant	IMPHY009490	Tricosane	
Crateva religiosa	whole plant	IMPHY009858	1-Undecanol	
Crateva religiosa	whole plant	IMPHY011215	Tetradecane	
Crateva religiosa	whole plant	IMPHY011396	4-Carvomenthenol	
Crateva religiosa	whole plant	IMPHY011939	10-epi-gamma-Eudesmol	
Crateva religiosa	whole plant	IMPHY012058	Linalool	
Crateva religiosa	whole plant	IMPHY012088	Mesitylene	
Crateva religiosa	whole plant	IMPHY012160	alpha-Terpineol	
Crateva religiosa	whole plant	IMPHY012654	Nerol	
Crateva religiosa	whole plant	IMPHY012920	2-Furanmethanol, 5-ethenyltetrahydro-alpha,alpha,5-trimethyl-, cis-	
Crateva religiosa	whole plant	IMPHY014923	Geraniol	
Crateva religiosa	whole plant	IMPHY014988	Limonene	
Crateva religiosa	whole plant	IMPHY014989	trans-Linalool oxide	
Crateva religiosa	whole plant	IMPHY015022	Nerolidol	
